# RAS-mediated tumor stress adaptation and the targeting opportunities it presents

**DOI:** 10.1242/dmm.049280

**Published:** 2022-02-11

**Authors:** Alexandra Redding, Andrew E. Aplin, Elda Grabocka

**Affiliations:** Department of Cancer Biology, Sidney Kimmel Cancer Center, Thomas Jefferson University, Philadelphia, PA 19107, USA

**Keywords:** RAS, Tumor-associated stress, RAS-pathway targeting, Drug resistance, Stress adaptation

## Abstract

Cellular stress is known to function in synergistic cooperation with oncogenic mutations during tumorigenesis to drive cancer progression. Oncogenic RAS is a strong inducer of a variety of pro-tumorigenic cellular stresses, and also enhances the ability of cells to tolerate these stresses through multiple mechanisms. Many of these oncogenic, RAS-driven, stress-adaptive mechanisms have also been implicated in tolerance and resistance to chemotherapy and to therapies that target the RAS pathway. Understanding how oncogenic RAS shapes cellular stress adaptation and how this functions in drug resistance is of vital importance for identifying new therapeutic targets and therapeutic combinations to treat RAS-driven cancers.

## Abstract (precis)

**Summary:** The acquisition of oncogenic RAS simultaneously causes cellular stress and activates stress-adaptive pathways. Many of these are implicated in resistance mechanisms to RAS-targeting therapies, posing as therapeutic targets to block stress-activated mechanisms of resistance.

## INTRODUCTION

The RAS pathway responds to external growth factors by activating genes that regulate several biological processes, including cell growth, division and differentiation. The pathway begins with the binding of growth factors to their cognate receptor at the cell surface, leading to the activation of the three isoforms of the small GTPase RAS (HRAS, KRAS and NRAS). RAS activation initiates multiple signaling cascades, which culminate in the activation of transcription factors, such as c-Myc (also known as MYC), c-JUN (also known as JUN), and ETS and CREB proteins (Chang et al., 2003). The hyperactivation of the RAS pathway due to the acquisition of activating mutations in RAS is an initiating event in malignant transformation; ∼19% of all cancer patients harbor an activating mutation in one of the RAS genes (Prior et al., 2020). As such, this prevalent oncogenic driver presents an opportune target in the treatment of a variety of cancer subtypes. However, inhibiting the RAS protein in a clinical context has proven challenging for a variety of reasons (Choi et al., 2019). These include its active site being tucked away deep inside the protein and thus being unavailable for small-molecule binding, its high affinity for GTP, and differences in the structure and hydrolysis rates among specific RAS mutants (Smith et al., 2013; Cagir and Azmi, 2019).

**Fig. 1.**
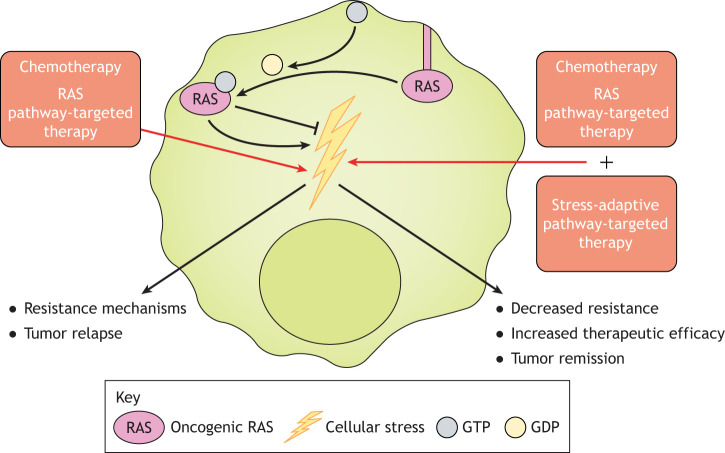
**Targeting stress-adaptive pathways to enhance chemotherapy and RAS pathway-targeted therapies.** Oncogenic RAS signaling can both induce cellular stress and inhibit such stress through the induction of stress-adaptive pathways, making cancer cells more tolerant to intrinsic and chemotherapy-derived stresses and thus providing them with a survival advantage. Upregulated stress-adaptive pathways might also contribute to acquired resistance mechanisms in RAS-driven cancer cells. When targeting a RAS-driven cancer cell, it may therefore be necessary to block such stress-adaptive pathways when providing the initial therapy, to reduce tolerance and to block the acquisition of stress-adaptive mechanisms. GDP, guanosine-5′-diphosphate; GTP, guanosine-5′-triphosphate.

Several studies have indicated that oncogenic RAS and cellular stress cooperate in driving tumorigenesis. Cell stress is a double-edged sword that promotes tumorigenesis but can also lead to cell death once a threshold is crossed. Oncogenic RAS is involved in the induction of a variety of cellular stresses, such as hypoxia (Kikuchi et al., 2009), metabolic stress (Fritsche-Guenther et al., 2018), oxidative and endoplasmic reticulum (ER) stress (Liou et al., 2016), and DNA damage and replication stress (Al Zubaidi et al., 2021; Maya-Mendoza et al., 2015), which all can promote tumorigenesis. RAS-driven tumorigenesis, however, is tightly linked to the activation of stress-adaptive mechanisms, which mitigate against the levels of these stresses (Alves et al., 2015; Denicola et al., 2011; Grabocka and Bar-Sagi, 2016). Therefore, RAS-driven, stress-adaptive mechanisms can be thought of as vulnerabilities, as the cancer cell depends on them to survive. As such, understanding how RAS activates these stress-adaptive pathways, and their role in tumorigenesis and therapy response, may lead to the discovery of novel vulnerabilities in RAS mutant cancer cells that can be targeted in the treatment of these tumors (Fig. 1).

The discovery of small molecules that bind to and inactivate KRAS^G12C^, a mutant form of RAS present in multiple cancer types, was an important breakthrough in the field, allowing for a direct inhibition of one form of aberrant RAS with a promising output in the clinic (Li et al., 2021a; Jänne et al., 2020; Canon et al., 2019; Cagir and Azmi, 2019). Nonetheless, MAPK-driven resistance mechanisms to KRAS^G12C^ inhibitors have already been identified, suggesting that the acquisition of resistance by tumors will challenge the efficacy of these inhibitors (Ryan et al., 2020; Xue et al., 2020; Tanaka et al., 2021). As such, combinatorial approaches that target mechanisms of resistance and RAS pathway regulators and effectors will likely be required for long-term, efficacious therapeutic response. Many factors contribute to the resistance mechanisms that cancers evolve, including tumor and tissue type, the surrounding microenvironment, the heterogeneous populations of cancer cells and the direct pressures of a specific therapy. As will be discussed, resistance mechanisms can be born out of stress-adaptive pathways (Fig. 1), as different nodes within such pathways have been implicated in acquired resistance mechanisms. This route of resistance may be heightened in cancers driven by oncogenic RAS, as their survival has been shown to be intricately interwoven with, and dependent upon, cellular stress responses. Not only can this potentiate acquired resistance, but the upregulation of stress-adaptive responses can also lead to a stress-tolerant phenotype in which cells have an enhanced survival edge at the start of therapy. In addition, because tumors consist of a heterogeneous pool of cancer cell populations, a range of cellular fitness can be present in a single tumor, which can be specific to a particular stress or environmental condition. Therefore, the problem of resistance in treating RAS-driven cancers is complex, but focusing on common themes of resistance, such as stress-adaptive pathways, may aid in the identification of widespread, RAS-driven routes of resistance.

In this Review, we describe the relationship between oncogenic RAS and various stress-adaptive pathways. In addition, we examine multiple stress-linked survival and resistance mechanisms present in RAS-driven tumors to better understand how oncogenic RAS operates as a primary inducer of stress, and in response to stress, to favor survival. These stress-adaptive mechanisms are pertinent to understanding therapeutic outcome in the clinic, as resistance is still a major setback when treating RAS-driven cancer. As new RAS pathway-targeting therapies arise, the investigation of therapy-induced stress adaptive pathways should be of great importance, as this may pinpoint appropriate targets that confer resistance for that specific therapy or cancer type.

## RAS-driven adaptations to cellular stress

Oncogenic RAS directly induces various kinds of cellular stress, and these stress-adaptive responses have been implicated in promoting tumorigenesis. However, because survival in the face of stress often relies on the duration and severity of such stress, oncogenic RAS also upregulates pathways that aid in stress mitigation. This section will describe how oncogenic RAS induces stress-adaptive tumor-promoting pathways and keeps them in check by upregulating other pathways that modulate the stress intensity.

### Adaptation to oxidative stress

Oxidative stress is defined as an imbalance in the levels of free radicals, and the inability to detoxify free radicals and their harmful effects. Heightened oxidative stress is a key feature of oncogenic RAS-driven cancers (Irani et al., 1997; Vafa et al., 2002). The increased formation of radical oxygen species (ROS), such as superoxide anion (O^2−^) and hydrogen peroxide (H_2_O_2_), is a common characteristic of cancer cells (Szatrowski and Nathan, 1991). ROS are generated via the electron transport chain, via the activation of NADPH oxidases (such as NOX1), or through the activity of lipoxygenases, among other mechanisms. As they are major modulators of cell signaling and gene expression, certain levels of ROS are necessary for cellular function (Chandel et al., 2000; West et al., 2011). However, ROS are also damaging agents that can interact with DNA and proteins, cause lipid peroxidation and lead to apoptosis (Wang et al., 2008). Oxidative stress is kept in check by the antioxidant program – an intrinsic mechanism by which cells maintain an appropriate level of free radicals that opposes the formation and activity of ROS. Antioxidant enzymes, such as superoxide dismutase (SOD), glutathione peroxidase (GPX), catalase and others, break down ROS into non-damaging molecules (Ighodaro and Akinloye, 2018). Scavenger molecules also readily react with ROS, reducing the interaction between ROS and cellular proteins through competition.

Oncogenic RAS increases ROS levels through a variety of mechanisms, including enhanced activity of NOX proteins, which produce superoxide. Specifically, RAS elevates ROS levels through MAPK-mediated transcriptional upregulation of Nox1 (Mitsushita et al., 2004), p38 (also known as MAPK11)-mediated stabilization and translocation of the p47^phox^ subunit of Nox1 (Park et al., 2014), and via COX-2 (also known as PTGS2)-mediated prostaglandin E production, which generates H_2_O_2_ as a byproduct (Maciag et al., 2004). In oncogenic RAS-driven human pancreatic tumors and in pancreatic cancer mouse models, the levels of the ROS-inducing NOX4 correlate positively with tumor progression, indicating a heightened reliance on ROS during tumorigenesis (Ogrunc et al., 2014). Oncogenic RAS and mutant BRAF also contribute to the antioxidant program, as their expression increases the transcriptional levels of Nrf2 (also known as NFE2L2), a transcription factor that binds to antioxidant response elements and promotes the expression of antioxidant genes (Denicola et al., 2011; Mukhopadhyay et al., 2020). This stress-adaptive mechanism is pro-tumorigenic, as *Nrf2*^−/−^ mouse models of pancreatic cancer have fewer pancreatic intraepithelial neoplasia (PanINs), which are also less proliferative and have higher levels of senescence compared to Nrf2-expressing counterparts (Denicola et al., 2011). The reduction in proliferation of *Nrf2*-deficient PanINs can be rescued by the addition of the antioxidant N-acetyl cysteine, indicating that this oncogenic RAS-driven antioxidant program contributes to PanIN formation and progression. In addition to supporting the antioxidant program, oncogenic RAS inhibits H_2_O_2_-induced apoptosis (Young et al., 2004). Together, these studies indicate that oncogenic RAS directly aids in the adaptation to the oxidative stress it induces, resulting in increased tumorigenesis *in vivo*.

### Adaptation to metabolic stress

Cancer cells have a higher demand for nutrients and energy relative to non-transformed cells, to support their rapid levels of growth and proliferation, and often become stressed in the attempt to satisfy these metabolic needs. As a cancer cell population grows, it faces poorer perfusion, leading to the decreased availability of oxygen, glucose and other nutrients in the microenvironment, further challenging such metabolic demands (Munir et al., 2019). In order to survive, cancer cells rewire their metabolism and rely on increased scavenging mechanisms to support their metabolic needs (Commisso et al., 2013; Kamphorst et al., 2015; Son et al., 2013; Humpton et al., 2019). These intracellular metabolic alterations include changes in amino acid metabolism (Wei et al., 2021), a shift from oxidative phosphorylation to glycolysis known as the Warburg effect (Liberti and Locasale, 2016; Vaughn and Deshmukh, 2008), enhanced lipogenesis (Munir et al., 2019) and alterations in metabolic enzymes, such as isocitrate dehydrogenase I and II (Reitman et al., 2011). Many of these changes are universal across cancer types, but the underlying mechanisms can be influenced by the oncogenic driver (Munir et al., 2019; Kamphorst et al., 2013). For example, oncogenic HRAS promotes elevated lipid uptake, but the expression of constitutively active myristoylated Akt promotes increased *de novo* lipid synthesis (Kamphorst et al., 2013). These metabolic changes have important implications for cancer progression. For example, the inhibition of key metabolic enzymes that are upregulated in cancer, such as lactate dehydrogenase A (LDH-A) and the hexokinase isoform HK2, delays tumor progression (Krushna et al., 2013; Liu et al., 2019). The glutaminase inhibitor CB-839, which targets an essential enzyme involved in glutamine metabolism, has shown promise in clinical trials, generating an objective response rate of 42% and a disease control rate of 100%, with 42% partial response and a 58% stable disease, when combined with the tyrosine kinase inhibitor cabozantinib in patients with metastatic renal cell cancer (Meric-Bernstam et al., 2019; https://clinicaltrials.gov/ct2/show/NCT02071862).

Oncogenic RAS influences metabolic stress directly through its translocation to the mitochondrial membrane and induction of mitochondrial dysfunction through the inhibition of complex I (Hu et al., 2012). Oncogenic RAS can also directly promote stress-adaptive processes that respond to metabolic stress, including increased macropinocytosis, autophagy and anabolic processes. Macropinocytosis, the actin-dependent process of extracellular fluid engulfment at the plasma membrane, is upregulated in human pancreatic ductal adenocarcinoma (PDAC) tissue and in KRAS^G12C^-mutant PDAC-derived human cells (Commisso et al., 2013; Kamphorst et al., 2015). One result of macropinocytosis is the internalization of extracellular proteins that can be degraded into amino acids, such as glutamine, on which cancer cells are metabolically dependent (Commisso et al., 2013; Kamphorst et al., 2015). The pharmacological inhibition of macropinocytosis in nude mice that were subcutaneously injected with MIA Paca-2 pancreatic cancer cells decreased tumor growth and even caused tumor regression in some animals (Commisso et al., 2013). The attenuation of tumor growth was specific to pancreatic tumors that express oncogenic KRAS, suggesting that macropinocytosis is a critical KRAS-induced survival mechanism (Commisso et al., 2013).

In addition to macropinocytosis, oncogenic RAS can supply nutrients to metabolically stressed cells by upregulating autophagy through the Rac1–JNK and the MEK–ERK (also known as MAP2K–MAPK) pathways (Byun et al., 2009; Wu et al., 2011). Autophagy is a process by which organelles and macromolecules are degraded into smaller molecules that can be re-used in metabolic pathways and is often activated as an adaptation to metabolic stress (Guo et al., 2011; Poillet-Perez et al., 2015; Meijer et al., 2015). The overexpression of wild-type or oncogenic KRAS proteins increases basal levels of autophagy (Alves et al., 2015). Under starvation, overexpression of oncogenic KRAS^G13D^ and KRAS^G12D^ in human non-cancerous colon cells increases levels of autophagy, suggesting that mutant KRAS plays a specific role in autophagy induction under stress (Alves et al., 2015). The suppression of autophagy in KRAS^G12V^-containing colorectal cancer patient-derived SW480 cells through the knockdown of *ATG5* and *BECN1*, which are involved in autophagosome formation, increases cell death during starvation, highlighting the importance of autophagy in nutrient stress adaptation (Alves et al., 2015).

A third mechanism by which oncogenic KRAS combats nutrient deprivation is through its effect on the expression levels of GOT1 and GLUD1, two major enzymes involved in glutamine metabolism (Son et al., 2013). Pancreatic cancer cells use GOT1 to fuel the citric acid cycle while maintaining the redox state of the cell (Son et al., 2013). KRAS knockdown in multiple PDAC cell lines increases the mRNA and protein levels of GLUD1 and decreases GOT1 (Son et al., 2013). This effect was mimicked *in vivo*, as *Got1* mRNA levels increased and *Glud1* mRNA levels decreased with the induction of KRAS expression in a pancreas-specific doxycycline-activated oncogenic KRAS-inducible mouse model. GOT1 knockdown led to an approximate sixfold reduction in tumor volume in this model, indicating that GOT1 aids in tumor growth (Son et al., 2013).

Oncogenic RAS also activates a selective mitophagy program that reduces mitochondrial ROS specifically, and redirects glucose metabolism away from the mitochondria through the increased expression of BNIP3L (also known as NIX) (Humpton et al., 2019). BNIP3L is a pro-apoptotic Bcl-2 family member, the interaction of which at the mitochondrial outer membrane promotes the entry of lysosomal proteins from the cytoplasm into the mitochondrial matrix, leading to mitophagy. Mitophagy involves the degradation of mitochondria through autophagy, often as a result of cellular stress or damaged mitochondria or, as in this instance, as an output of oncogenic signaling. RAS-driven mitophagy leads to a reduction in mitochondrial content. It also leads to a BNIP3L-dependent decrease in mitochondrial glucose flux and citric acid cycle intermediates, changes that indicate a channeling of glucose into aerobic glycolysis or into other anabolic processes (Humpton et al., 2019). This altered metabolism was hypothesized to lead to an increased survival advantage for RAS-driven tumors, as BNIP3L depletion via siRNA reduced the proliferation of KRAS^G12D^-expressing murine embryonic fibroblasts. In support of this, the conditional deletion of *Bnip3l* in pancreata of KC (KRAS^G12D^-expressing) and KPC (KRAS^G12D^- and p53^R172H^-expressing) mouse models resulted in lower-grade PanINs, as well as an increase in median survival (Humpton et al., 2019). Therefore, RAS not only induces metabolic stress, but also contributes to multiple mechanisms that promote survival during metabolic stress.

### Adaptation to ER stress

ER stress refers to an increased presence of unfolded proteins within the ER, which can arise from a variety of cancer-related insults, including hypoxia, oxidative stress, genomic instability, and enhanced protein production and secretion (Yang et al., 2014; Oakes, 2020). ER stress leads to the activation of the unfolded protein response (UPR), which can be both cytoprotective and cytotoxic, depending upon how well it can mitigate the accumulation of unfolded proteins. The UPR contains three main signaling nodes: inositol-requiring protein 1 (IRE1; also known as ERN1), activating transcription factor (ATF)-6, and PKR-like endoplasmic reticulum kinase (PERK; also known as EIF2AK3) (Yadav et al., 2014). Upon accumulation of misfolded proteins, these ER transmembrane proteins respond by activating signaling cascades that promote transcriptional and translational changes, such as the transient re-localization of specific classes of mRNAs from the ER into the cytoplasm (Reid et al., 2014), which integrate to favor either a return to homeostasis or the induction of apoptosis (Kadowaki and Nishitoh, 2013; Acosta-Alvear et al., 2007; Hetz et al., 2006).

**Fig. 2.**
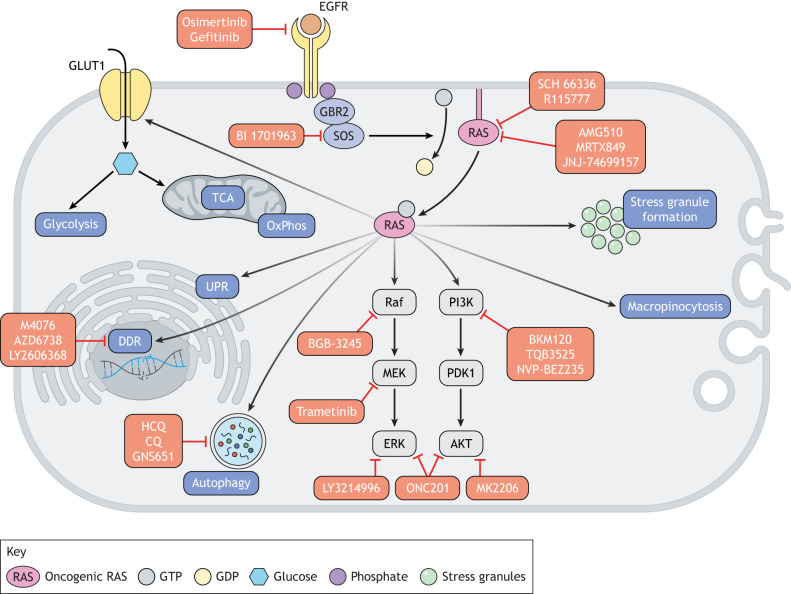
**Oncogenic RAS-driven induction of stress-adaptive mechanisms and current therapies along the signaling axis.** Oncogenic RAS induces multiple stress-adaptive pathways, such as altered (glucose) metabolism, UPR, DDR, autophagy, macropinocytosis and stress granule formation. Canonical oncogenic RAS signaling, such as the activation of the MEK–ERK1/2 and PI3K–AKT pathways, which directly promote proliferation, is also displayed. There are multiple drugs in clinical trials that target different nodes within these stress-adaptive and canonical pathways, as shown in red. There are still mutant RAS-driven stress-adaptive pathways that have yet to be targeted in the clinic, such as the formation of stress granules. CQ, chloroquine; DDR, DNA damage response; HCQ, hydroxychloroquine; OxPhos, oxidative phosphorylation; TCA, tricarboxylic acid cycle; UPR, unfolded protein response.

The UPR plays a significant role in cancer, as markers of this process are increased or altered across several cancer types (Fig. 2) (Yadav et al., 2014). In addition, the genetic ablation of *Ire1a* in intestinal epithelia-specific *Ire1a*-knockout mice (Li et al., 2017) and the mammary gland-specific knockout of *Perk* in mammary tumor-prone MMTV-*Neu* mice (Bobrovnikova-Marjon et al., 2010) reduce cancer growth and initiation, respectively, and the expression of PERK has been linked to chemoresistance in colon cancer cells and in subcutaneous xenograft models of colon cancer in NOD/SCID mice (Shi et al., 2019). The UPR pathway is upregulated in a variety of RAS mutant cancers and along the axis of RAS-driven tumor progression (Blazanin et al., 2017; Denoyelle et al., 2006; Catanzaro et al., 2014). In human and murine tissue samples of oncogenic RAS-driven acinar-to-ductal metaplasia (ADM) and PDAC, the ER stress-sensing protein GRP78 (also known as HSPA5) is upregulated in ADM and PDAC lesions, whereas little to no GRP78 is detected in corresponding wild-type samples (Hill et al., 2012). GRP78 disassociates from the ER transmembrane proteins IRE1, PERK and ATF6 under ER stress, leading to their dimerization and activation, which promotes UPR. In terms of tumorigenicity, GRP78 also contributes to various stem-like properties of pancreatic cancer cells, such as clonogenicity, self-renewal and invasion, which translate into a reduced capacity to initiate tumor formation and to decreased tumor weight in nude mice subcutaneously injected with pancreatic cancer cells (Dauer et al., 2019). Although this evidence supports the pro-tumorigenic properties of ER stress, it is important to note that it can also be anti-tumorigenic based upon severity and duration, which can explain the dual nature of oncogenic RAS in inducing or limiting the UPR (Maurel et al., 2015).

Consistent with the pro-tumorigenic role of ER stress, oncogenic RAS can directly impact ER stress levels through the activation of IRE1a via the MEK–ERK pathway (Blazanin et al., 2017). The expression of oncogenic HRAS in primary murine keratinocytes increases *Ire1a* mRNA and protein levels and its phosphorylation, indicative of the overall IRE1a activation (Blazanin et al., 2017). In addition, oncogenic RAS induces ER stress indirectly through ROS induction (Park et al., 2014; Mitsushita et al., 2004). As discussed above, ER stress can result in both cell survival and cell death, and tumorigenic progression requires the tempering of and/or adaptation to ER stress. IRE1a activation by oncogenic RAS results in the splicing of X-box-binding protein 1 (Xbp1), which has been implicated in stress adaptation during the UPR (Blazanin et al., 2017; Hollien et al., 2009). ER stress favors this particular activity of IRE1a, which was shown to be necessary for mutant HRAS-expressing cells to proliferate. By contrast, reduced ER stress favors senescence despite the retention of activated IRE1a. This shows that both the presence of ER stress and the activation of IRE1a through oncogenic RAS work together to promote a proliferation-supportive phenotype. Oncogenic RAS also specifically upregulates proteins that limit the ER stress response. In patient-derived myeloma cell lines that were engineered to constitutively express mutant forms of either KRAS, NRAS or BRAF, the expression of each of these oncogenes increased the transcription of proteasome 20S subunit beta 8, 9 and 10 (PSMB8, PSMB9 and PSMB10) (Shirazi et al., 2020). The transcriptional levels of the assembly chaperone proteasome maturation protein (POMP) and its upstream regulator Nrf2, which are required for the cleavage and activation of PSMB8/9/10, were also increased after the expression of the *KRAS*, *NRAS* or *BRAF* oncogenes (Shirazi et al., 2020). These results suggest that oncogenic RAS and BRAF may enhance proteasome capacity, which could mitigate the activation of the ER stress response through a reduction in proteotoxic stress. Surprisingly, the expression of these oncogenes also reduced the transcription of ATF4 and ATF6, which are involved in the ER stress response, showing that oncogenic RAS can directly dampen ER stress signaling as well.

### Adaptation to hypoxia

Hypoxia describes a state of low or inadequate oxygen availability, and can exist at the cell, tissue or organ level (Muz et al., 2015). It often occurs as a result of reduced blood flow to a particular region or because of the increased proliferation of cells within a tissue, such as in a tumor, where highly proliferative cancer cells consume more oxygen than normal cells and eventually outgrow their initial supply. Cells undergoing hypoxia respond by stabilizing hypoxia inducible factor 1 subunit alpha (HIF-1α), a transcription factor responsible for the activation of multiple genes involved in metabolism and angiogenesis, and vascular endothelial growth factor (VEGF; also known as VEGFA), which promotes angiogenesis to increase blood supply (Forsythe et al., 1996). Cancer cells are notorious for generating a dysfunctional vasculature through their stimulation of angiogenesis. Different isoforms of VEGF exist, and their activation can lead to differential vascularization patterns within a tumor (Yu et al., 2002). As this vascularization changes, the distinct spatial regions of a tumor experience periods of hypoxia and normoxia, leading to environmental pressures that select for cells that can survive under such conditions. In addition, hypoxia can induce epithelial-to-mesenchymal transition, which enhances the invasive and metastatic properties of cancer cells (Muz et al., 2015). Different organs across the body have varying levels of oxygen that are considered physiological, and each of these tissues experiences a specific drop in oxygen levels when a tumor is present (Muz et al., 2015). Therefore, in addition to the size of a tumor, the specific tissue it forms in can affect the extent of hypoxia.

Oncogenic RAS-driven tumors experience hypoxic conditions for the reasons described above, as oncogenic RAS increases cell proliferation. There are also a variety of mechanisms that link oncogenic RAS to the stress-adaptive mechanisms involved in cell survival during hypoxia. For example, the expression of oncogenic KRAS enhances HIF-1α function, and that of oncogenic BRAF enhances HIF-1α and HIF-2α (also known as EPAS1) function during hypoxia (Kikuchi et al., 2009). Receptor for advanced glycation end products (RAGE; also known as AGER), a protein primarily involved in inflammation, acts as a positive regulator of HIF-1α through its binding to oncogenic RAS during hypoxia (Kang et al., 2014). This binding is increased in human pancreatic cancer cells that express oncogenic RAS compared to a pancreatic cancer cell line that expresses wild-type RAS, suggesting that the mutational status of RAS may play a role in such binding (Kang et al., 2014). When MEK1/2 and AKT are inhibited in a murine pancreatic tumor cell line, RAGE can no longer activate HIF-1α, indicating that RAGE activates oncogenic RAS signaling to promote adaptation to hypoxic conditions (Kang et al., 2014). Moreover, knocking down RAGE in murine pancreatic tumor cell lines under hypoxia and knocking it out in KC mice *in vivo* reduces phospho-AKT and phospho-ERK1/2 levels (Kang et al., 2014). In addition to the activation of HIF-1α, oncogenic RAS has been shown to converge on a hypoxia-induced, stress-adaptive pathway that targets the tumor suppressor reversion-inducing cysteine-rich protein with Kazal motifs (RECK). RECK is a glycoprotein that downregulates matrix metalloproteinases that degrade extracellular matrix (ECM) proteins and contribute to tumorigenesis. RECK is inhibited during hypoxia through the activation of HIF-1α and miR-372/373 (Loayza-Puch et al., 2010). Oncogenic RAS contributes to this RECK inhibition through the upregulation of miR-21, potentially strengthening this response or priming the cell for survival during hypoxia (Loayza-Puch et al., 2010). Therefore, oncogenic RAS equips the cell to deal with a hypoxic environment, most notably by stabilizing HIF-1α and by converging on stress-adaptive pathways that inhibit the tumor suppressor RECK.

### Adaptation to biomechanical stress

In order to survive, cells must be able to physically sense their microenvironment and to adapt to changes or respond to signals within that environment. There are a multitude of biomechanical sensing molecules that integrate these external signals into cellular responses, including cytoskeletal proteins, adhesion receptors and ion channels (Daniel et al., 2013; Yao et al., 2014; Lim et al., 2018). These sensing mechanisms can control cell shape, stiffness, motility, proliferation, survival and fate in response to what they sense within the surrounding environment. Changes in the biomechanical sensing mechanisms of cells, as well as changes in tension and homeostasis within a tissue overall, can be initiating events in tumorigenesis (Fernández-Sánchez et al., 2015; Razzaghi et al., 2012; Beverly et al., 2005; Pan et al., 2020).

Oncogenic RAS plays a role in the biomechanical properties of cells, and assists with cell survival in a physically changing microenvironment, such as during mitotic rounding and in responses that involve cellular stiffness (Matthews et al., 2020; Lin et al., 2015). For example, the expression of oncogenic HRAS in Madin-Darby canine kidney-derived epithelial cells and in mouse mammary gland epithelial cells, and the overexpression of oncogenic KRAS in human pancreatic ductal cells, result in cell softening compared to parental cell lines *in vitro* (Lin et al., 2015). In addition, the proliferative capacity of cancer cell lines with oncogenic KRAS was less affected than that of normal cells when challenged with soft matrix growth conditions, suggesting that oncogenic RAS can promote adaptation to biomechanical stress by modulating cell stiffness (Lin et al., 2015).

Oncogenic RAS has also been shown to directly affect the composition of the microenvironment, aiding in both cancer cell survival and metastasis. Transformation with oncogenic RAS leads to the overexpression of tenascin-C, an ECM molecule that can drive cancer progression (Maschler et al., 2004; Sun et al., 2018, 2019). Oncogenic RAS also promotes survival during ECM detachment (Mason et al., 2016). ECM detachment induces metabolic stress and the cell death program, anoikis, in normal cells, but is an initiating step in the metastasis of cancer cells (Mason et al., 2016; Schafer et al., 2009). When oncogenic RAS-expressing cells undergo ECM detachment, RAS blocks anoikis via the activation of serum/glucocorticoid-regulated kinase 1 (SGK-1) and the downregulation of PH domain leucine-rich repeat protein phosphatase (PHLPP; also known as PHLPP1), which inhibits the activation of the p38 MAPK pathway and blocks its role in anoikis, thus promoting survival and supporting metastasis (Mason et al., 2016). Therefore, oncogenic RAS mediates the stiffness-sensing mechanisms of the cell, affects the matrix of the surrounding microenvironment and favors cell survival during metastasis.

### Adaptation to pan-stress stimuli

As described thus far, RAS-driven cancer cells are exposed to a range of cellular stresses, and oncogenic RAS can respond to these stresses in different ways by upregulating specific, stress-adaptive mechanisms. Oncogenic RAS can also respond to multiple stresses to enhance the overall stress tolerance of a cell, and these mechanisms can be considered adaptations to pan-stress stimuli. One of the major oncogenic, RAS-driven, pan-stress adaptations is the upregulation of stress granules (SGs) (Fig. 2). SGs are non-membranous cytoplasmic organelles that consist of protein and RNA and that assemble in response to various stress stimuli, such as hypoxia (Arimoto et al., 2008; Gottschald et al., 2010), oxidative stress (Namkoong et al., 2018), DNA damage (Byrd et al., 2016; Moutaoufik et al., 2014) and ER stress (Namkoong et al., 2018). SGs confer cytoprotection and promote survival, as evidenced by the fact that blocking SG formation under stress reduces cell survival in human breast and colon cancer cells *in vitro* (Arimoto et al., 2008; Grabocka and Bar-Sagi, 2016). SGs can directly oppose apoptosis by reducing ROS levels, through the sequestration of mammalian target of rapamycin complex 1 (mTORC1) via the spindle-associated protein astrin, and through the sequestration of RACK1, a scaffolding protein involved in the stress-activated MAPK-driven apoptotic response (Arimoto et al., 2008; Takahashi et al., 2013; Thedieck et al., 2013). Proteins that modulate SG assembly are upregulated in many human cancer types, including cancer, colorectal and prostate cancer, and sarcoma, and their expression levels often correlate with a poorer prognosis in the patient (Somasekharan et al., 2015; Sim et al., 2019; Li et al., 2020; Wang et al., 2021). *In vivo*, SGs have been implicated in metastasis, as osteosarcoma cells with knockdown of G3BP1 were associated with reduced levels of lung metastases upon implantation in the kidney capsule, compared to control osteosarcoma cells, which formed lung metastases within 4-5 weeks of implantation (Somasekharan et al., 2015).

Gain- and loss-of-function experiments in pancreatic and colorectal cancer cell lines demonstrate that oncogenic KRAS promotes SG formation as an adaptive mechanism to a variety of tumor-associated stress stimuli (Grabocka and Bar-Sagi, 2016). The induced expression of mutant HRAS also increases the SG-forming capacity of cells, suggesting that this phenotype may translate across mutant RAS isoforms (Grabocka and Bar-Sagi, 2016). Oncogenic KRAS-mediated SG assembly depends on the production of the lipid-signaling molecule 15-d-PGJ2, which occurs via the RAS–ERK-mediated regulation of two key enzymes, COX-2 and 15-hydroxyprostaglandin dehydrogenase (HPGD) (Grabocka and Bar-Sagi, 2016; Qiang et al., 2019). A particularly interesting aspect of this oncogenic RAS-induced stress response is that it can occur in a cell non-autonomous manner via the secretion of 15-d-PGJ2 (Grabocka and Bar-Sagi, 2016). Therefore, not only does oncogenic RAS enhance stress tolerance in the cell in which it operates, but it might also enhance the fitness of the surrounding cells in the microenvironment (Grabocka and Bar-Sagi, 2016). SGs might thus be a powerful RAS-induced stress-adaptive mechanism, as they are a singular output that responds to multiple challenges that a RAS-transformed cell faces. There is more to be uncovered about how SGs function in the different stages of tumorigenesis and about the specific mechanisms by which these granules combat the different stresses. Deriving answers to these questions would constitute an important step forward for the field, as such knowledge might aid in the identification of therapeutic targets that could hinder this pan-stress adaptation mechanism.

### Stress adaptation in the persister cell phenotype

It is clear that multiple stress-adaptive pathways activated by oncogenic RAS can promote survival in the face of transformation-related stress. However, the way in which oncogenic RAS prepares the cell to deal with external stress, such as from the tumor microenvironment or chemotherapy, is also of great importance. One of these RAS-driven stress-adaptive pathways has recently been implicated in generating a stress-tolerant cell state, called a cycling persister cell. Tolerant cells are described as cells that have a reduced sensitivity to a particular drug or stress, whereas ‘persisters’ are cells that can enter into a dormant state to survive a particular drug or stress (Sharma et al., 2010; Kurppa et al., 2020). Although most persister cells remain dormant throughout a treatment, some can re-enter the cell cycle during treatment, and thus pose an immediate threat to a positive therapeutic outcome (Oren et al., 2021). Cellular programs that contribute to a cycling, persister cell phenotype during treatment have been described. The antioxidant program's genes have been shown to be more highly expressed in cycling persister cell clones and to be targets of Nrf2 (Oren et al., 2021). When ROS levels are reduced in persister cells, through treatment with the scavenger molecule NAC or via the overexpression of the antioxidant enzyme glutathione peroxidase 2, the fraction of cycling persister cells increases by sixfold and threefold, respectively. This finding indicates that the activation of the antioxidant program might support the re-entry of persister cell populations into the cell cycle (Oren et al., 2021). Interestingly, oncogenic RAS increases ROS and Nrf2 expression levels, raising the possibility that RAS might contribute to the cycling persister cell population through this mechanism. The role of oncogenic RAS in promoting the emergence of persister cells and the ability of these cells to re-enter the cell cycle are important questions for future research to address.

Overall, oncogenic RAS is apt at providing survival mechanisms to combat its own stress induction, and may also contribute to an overall stress-tolerant phenotype that promotes endurance in the face of external stresses, such as chemotherapy. The following sections will describe how several resistance mechanisms are borne out of these oncogenic RAS-driven, stress-adaptive pathways, suggesting that oncogenic RAS also functions on the axis of external stress and chemoresistance.

## Stress adaptation in drug tolerance and tumor resistance

Unbiased drug screens for synthetic lethality and for other multifaceted vulnerabilities of mutant RAS-driven cancer cells have identified specific stress response proteins and entire stress-adaptive pathways that, when inactivated, lead to increased cell death, decreased tumorigenesis and decreased tumor progression (Yang et al., 2019; Elliott et al., 2019; Luo et al., 2009). Findings from these screens support the idea that stress-adaptive responses are key contributors to the survival and resistance mechanisms of RAS mutant cancer cells, and they provide evidence that the targeting of these pathways can overcome resistance to the targeting of RAS itself and of RAS pathway components. In this section, we describe how the inhibition of stress-adaptive pathways might challenge some of the current clinical problems concerning resistance and we consider how therapy itself might induce particular stresses that lead to novel stress-response vulnerabilities in these tumors. Of note, oncogenic RAS cells also utilize adaptive mechanisms to promote resistance to conventional chemotherapeutics, which rely largely on DNA damage. The role of oncogenic RAS in inducing the DNA damage response (DDR), in promoting adaptation to DDR (Fig. 2), and the therapeutic strategies for targeting this stress response have been reviewed in detail elsewhere and are summarized in Table 1 (Grabocka et al., 2015; Reid et al., 2014; Li et al., 2021b). Some characteristics of RAS mutant cells that are pertinent to the response to classic chemotherapy and the reported resistance mechanisms that utilize the cellular stress response are summarized in Table 2.

**Table 1.**
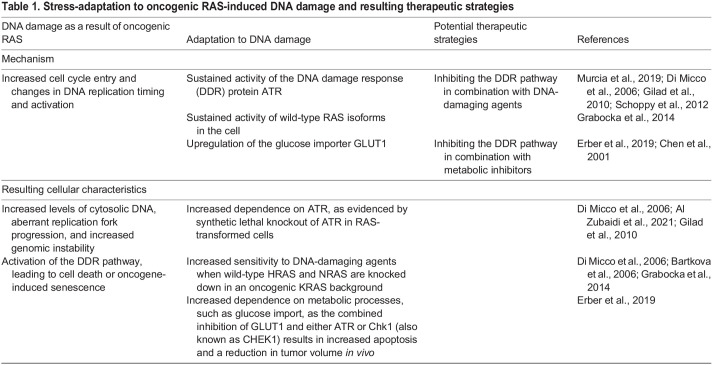
Stress-adaptation to oncogenic RAS-induced DNA damage and resulting therapeutic strategies

**Table 2.**
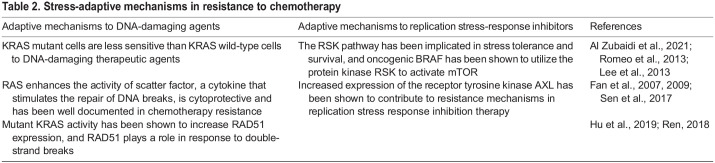
Stress-adaptive mechanisms in resistance to chemotherapy

### Autophagy in drug response

The survival and progression of RAS mutant tumors has a complex relationship with autophagy, and the dependence of these tumors on autophagy has been well documented (Fig. 2) (Guo et al., 2011; Poillet-Perez et al., 2015; Guo et al., 2016; Lock et al., 2014). Autophagy-related 7 (ATG7) regulates autophagosome formation and is required for autophagy to occur. In an oncogenic KRAS-driven non-small cell lung cancer (NSCLC) mouse model, deleting *Atg7* specifically in tumor cells reduced the tumor burden compared to that in mice with Atg7-expressing NSCLC. In addition, these Atg7-deficient adenomas progressed into oncocytomas as opposed to the adenocarcinomas seen in Atg7-expressing mice (Guo et al., 2013). An attractive clinical strategy has been to inhibit autophagy in such tumors; however, these monotherapies have ultimately failed due to sustained disease progression (Wolpin et al., 2014). More recently, data supporting the combined inhibition of autophagy and various proteins in the oncogenic RAS pathway has brought autophagy back into the spotlight (Bryant et al., 2019; Kinsey et al., 2019; Leung et al., 2018; Lee et al., 2019). For example, a recently identified small molecule, deltarasin, can disrupt the association of the chaperone phosphodiesterase-δ (PDEδ) with RAS, preventing PDEδ-mediated recruitment of RAS to the plasma membrane and therefore its activation (Leung et al., 2018). Deltarasin alone has a strong impact on tumor weight in a lung cancer cell xenograft mouse model; the average tumor weight of deltarasin-treated mice was 57% less than that of vehicle-treated controls (Leung et al., 2018). This reduction in tumor size is due to the induction of apoptosis caused by deltarasin-mediated PDEδ inhibition. However, deltarasin treatment also leads to protective autophagy, indicating that blocking autophagy might enhance the efficacy of deltarasin (Leung et al., 2018). In support of this, the anti-autophagic drug 3-MA more effectively induces cancer cell death through apoptosis when combined with deltarasin *in vitro* than when the cells are treated with deltarasin alone (Leung et al., 2018). Similar results have also been reported when autophagy inhibitors are combined with ERK1/2 inhibitors (ERKi) in patient-derived pancreatic cancer xenograft models and when used in triple combination with the BRAF and CRAF (also known as RAF1) kinase inhibitors in KRAS-mutant cell lines (Bryant et al., 2019; Lee et al., 2019). For example, one study has shown that treating KRAS-mutant pancreatic cancer patient-derived xenograft models with ERKi alone decreased tumor weight twofold compared to that of vehicle-treated controls. By contrast, when ERKi were combined with the autophagy inhibitor hydroxychloroquine, they reduced tumor weight by approximately sixfold (Bryant et al., 2019). These results indicate that, although oncogenic RAS induces autophagy, inhibiting the RAS pathway can also lead to stress-adaptive autophagy. As such, pairing RAS pathway inhibitors with autophagy inhibitors might push a cancer cell towards programmed cell death instead of towards tumor-promoting autophagy responses.

In addition to the discovery that RAS pathway inhibition leads to protective autophagy, new insights into the cellular response to autophagy inhibitors have also come to light. A comprehensive pharmacological screen recently identified replication response inhibitors and the lysosome inhibitor chloroquine (CQ) as inducers of synthetic lethality in PDAC cells (Elliott et al., 2019). CQ has long been used to target lysosomal pathways, and inhibits the final stage of the autophagy response. This study revealed that reduced nucleotide biosynthesis in response to CQ treatment leads to replication stress, rendering the cells vulnerable to replication stress inhibitors. This phenotype was partially rescued by supplementation with aspartate, a precursor for *de novo* nucleotide synthesis (Elliott et al., 2019). These findings support the notion that commonly used drugs, such as autophagy inhibitors and membrane localization inhibitors of RAS, which have failed as monotherapies against RAS-driven cancers, may induce particular stress-adaptive responses that aid in a cancer's survival and resistance to such therapies. Identifying these secondary stress responses may thus expose new targetable vulnerabilities while blocking such responses may revive old therapeutic strategies.

### Macropinocytosis in drug response

Oncogenic RAS can be a potent inducer of macropinocytosis, depending on the type of oncogenic mutation involved (Fig. 2) (Hobbs et al., 2020). However, the KRAS^G12R^ mutant, which is rare in lung and colorectal cancer but more common in pancreatic cancer, is dispensable for the characteristic upregulation of macropinocytosis, as shown from the examination of ten different PDAC cell lines (Hobbs et al., 2020). This mutation causes a structural change in the protein that renders it incapable of binding to PI3Kα. A distinct PI3K isoform, p110γ (also known as PIK3CG), compensates for this loss and is responsible for the KRAS-independent upregulation of macropinocytosis in these cells (Hobbs et al., 2020). KRAS^G12R^ mutant cells are also more sensitive than KRAS^G12D/V^ cells to MEK/ERK and PI3Kγ inhibition (Hobbs et al., 2020). This increased sensitivity to PI3Kγ inhibition is most likely due to the inability of KRAS^G12R^ to activate the PI3K pathway, indicating that different RAS mutations might require specific therapeutic strategies to effectively target stress-adaptive pathways. Future work should include the investigation of compensatory mechanisms that result from structural and functional differences between RAS mutant subtypes, as this may help to lead to more individualized and effective treatments for RAS-driven cancers.

Although RAS mutants might employ different mechanisms to upregulate macropinocytosis, it is nevertheless elevated in most RAS-driven cancers. One idea, therefore, is to use this enhanced macropinocytosis as a system for delivering drugs, rather than trying to inhibit it (Liu and Ghosh, 2019). RAS mutant cancer cells preferentially scavenge lipids, glutamine and albumin through macropinocytosis (Liu and Ghosh, 2019). For example, cross-linked albumin nanoparticles are taken up in greater quantities by cells with oncogenic KRAS than by their wild-type counterparts, and colocalize with macropinosomes, indicating that macropinocytosis was the uptake mechanism (Liu and Ghosh, 2019). This system might therefore be used in the future to deliver drugs selectively to KRAS mutant cells, potentially reducing toxicity to non-transformed cells and enhancing treatment efficacy.

Macropinocytosis aids in cancer anabolism and can directly enhance resistance to anabolism-targeting therapies (Jayashankar and Edinger, 2020). Anabolism is the biosynthesis of macromolecules that support the metabolic needs of cells, and common therapies that target anabolism include gemcitabine, 5-fluorouracil (5-FU), doxorubicin and γ-irradiation (Jayashankar and Edinger, 2020). These drugs often kill cancer cells via necrosis, which is a sudden and pro-inflammatory form of cell death in which the contents of the dying cell are released into the surrounding environment. When surrounding cells undergo necrosis within the tumor microenvironment, RAS-mutant cancer cells use macropinocytosis to take up the macromolecular end products that form in the cellular debris to boost their nutrient supply. The presence of such debris can also reduce the sensitivity of macropinocytic oncogenic RAS cells to anabolism-targeting therapies, as seen in oncogenic pancreatic cancer cells, which lose their sensitivity to 5-FU when it is added alongside necrotic cellular debris. These cells showed proliferation levels similar to those of their untreated counterparts, whereas non-RAS mutant with low macropinocytosis cells remained sensitive to 5-FU. 5-[N-ethyl-N-isopropyl] amiloride (EIPA) is a Na^+^/H^+^ exchanger inhibitor that blocks macropinocytosis without affecting receptor-mediated endocytosis. When cells were treated with 5-FU in the presence of necrotic cell debris and EIPA, the aforementioned survival advantage of RAS mutant cells was lost, indicating that necrotic cellular debris uptake had occurred via macropinocytosis (Jayashankar and Edinger, 2020). As the macropinocytosis-mediated uptake of macromolecules renders highly macropinocytic cancer cells tolerant to anabolic-targeting therapies, therapies that target both macropinocytosis and anabolic metabolism might provide a promising combination by which to block resistance mechanisms that emerge in the presence of anabolic-targeting therapies.

### ER stress adaptation in drug response

In support of the importance of the UPR stress response in cancer cell stress tolerance and drug resistance, a recent drug-screening study identified inhibitors of heat shock protein 90 (HSP90) proteins and AXL as the most detrimental to the growth of chemoresistant/MEK inhibitor-resistant cell lines, compared to therapy-naïve parental control cell lines (Yang et al., 2019). HSP90 proteins are chaperones responsible for proper protein folding, trafficking and degradation, and are involved in regulating the UPR response. AXL is a receptor tyrosine kinase that has been shown to activate the RAS pathway. Inhibitors of HSP90 and MEK, when combined, have strong anti-tumor effects in KRAS-mutant lung cancer patient-derived xenograft mouse models and in NSCLC xenograft mouse models, showing a three- to fourfold reduction in tumor weight compared to that of vehicle-treated controls (Yang et al., 2019). HSP90 inhibition has also been shown to preferentially induce apoptosis in KRAS-mutant colon cancer cells *in vitro* and in a colon cancer-derived xenograft model in nu/nu mice, indicating that this vulnerability might translate across different RAS-mutant tumor types (Wang et al., 2016). Targeting the UPR pathway can also block another stress-adaptive mechanism, protective autophagy, and can overcome resistance in melanoma cell lines, making the blocking of the UPR stress response an even more attractive approach (Ma et al., 2014). In the first example, protective autophagy was induced in response to BRAF inhibitors and blocked by the addition of a PERK inhibitor, leading to increased cell death. These findings suggest that blocking the UPR stress response might be an effective way to overcome this resistance mechanism (Ma et al., 2014). The same study also shows how stress-adaptive pathways are often interlinked, and how identifying and targeting the most critical mechanism for a cell could reduce its overall stress tolerance. In addition, it might be possible to identify which patients would most benefit from UPR-based combinatorial therapies by assessing their levels of UPR activity (Yang et al., 2019). Thus, stress-adaptive pathways could be used as biomarkers to predict patient responses to specific stress-targeting therapies and to predict which resistance mechanisms might emerge by profiling the stress-adaptive responses that are already heightened at the start of treatment.

### Adaptation to pan-stress stimuli in drug response

As previously described, oncogenic KRAS signals upregulate SG formation via the production of the signaling molecule 15-d-PGJ2. This process promotes survival in response to a variety of RAS- and chemotherapy-induced stresses. For example, in oncogenic KRAS-expressing HeLa cells, levels of oxidative stress-induced apoptosis increased following the addition of the SG inhibitor emetine (Grabocka and Bar-Sagi, 2016). By contrast, apoptosis levels in wild-type HeLa cells remained unaffected by emetine treatment, indicating that SGs play a specific role in survival during stress in oncogenic KRAS-driven cells (Grabocka and Bar-Sagi, 2016). When SG formation was blocked using a COX-1/2 inhibitor in oncogenic KRAS-driven colon cancer cells, the cells also showed increased sensitivity to the chemotherapeutic drug oxaliplatin. This effect functioned at a paracrine level, consistent with the paracrine induction of SGs by oncogenic KRAS (Grabocka and Bar-Sagi, 2016). Multiple anti-cancer drugs have been shown to induce SGs, including 5-FU, lapatinib, sorafenib, oxaliplatin, bortezomib and selenite, to name a few (Kaehler et al., 2014; Adjibade et al., 2020; Hu et al., 2021). One study reported that 5-FU treatment of colorectal cancer cell lines *in vitro* increased their expression of Musashi-1, a colon stem cell marker and RNA-binding protein, which contributed to the formation of anti-apoptotic SGs and to the population of CD44^+^ stem cells (Chiou et al., 2017). In ovarian carcinoma cells, the inhibition of Musashi-1 blocked paclitaxel resistance, implicating this SG-promoting protein in drug resistance (Chen et al., 2019). Given that SGs are a mechanism of resistance induced by both oncogenic KRAS and chemotherapy, with the former also creating resistance in a paracrine manner in surrounding tissue, SG-targeting agents are likely to provide potent therapeutics for treating oncogenic RAS-driven tumors.

Other proteins or pathways might also respond to a multitude of RAS-induced stresses. One such example has been identified in the investigation of resistance mechanisms that accompany treatment with EGFR inhibitors. Oncogenic RAS colorectal cancer cells that are sensitive to EGFR-targeting antibodies undergo apoptosis through the p73-dependent transcriptional activation of the BH3-only protein PUMA (also known as BBC3); when these cells acquire resistance, they exhibit a reduction in *PUMA* expression (Knickelbein et al., 2018). PUMA induces apoptosis in response to ER and genotoxic stress, and to deregulated oncogenic signaling (Yu and Zhang, 2008). Thus, PUMA loss might be a stress-adaptive mechanism that promotes survival in the context of many oncogenic RAS-induced stresses. The reactivation of PUMA, when combined with RAS pathway inhibition, might produce a synergistic effect that promotes apoptosis and reduces the survival of RAS-driven tumor cells. Another potential strategy would be to induce PUMA alongside inhibiting autophagy, because autophagy protects against many oncogenic RAS-induced stresses. Overall, it is apparent that some oncogenic RAS-induced, and therapy-derived, stress-adaptive mechanisms lead to a stress-tolerant state that mitigates against a plethora of RAS-induced stresses. As such, the identification and targeting of such mechanisms might be the most effective way to enhance the efficacy of RAS-targeted therapies.

### Emerging resistance to KRAS^G12C^ inhibitors

The mechanisms that underlie resistance to KRAS^G12C^ inhibitors in lung and other types of cancer are at an early stage of investigation. Thus far, a variety of resistance mechanisms have been described, but most seem to share the common end result of reactivating the MAPK pathway (Ryan et al., 2020; Xue et al., 2020; Tanaka et al., 2021), such as acquired mutations in BRAF, NRAS, MAP2K1 (MEK1) and KRAS itself (Tanaka et al., 2021). These acquired KRAS mutations include other common variants in KRAS that are seen across mutant KRAS-driven cancers, such as the G13D and G12V substitutions, as well as a novel mutation in residue 96 (KRAS^Y96D^) that are yet to be documented in the clinic (Tanaka et al., 2021). Unfortunately, many of these acquired resistance mechanisms were identified from the biopsies of a single patient, indicating that resistance to KRAS^G12C^ inhibitors is quite heterogeneous. This would suggest that there may be an even greater level of heterogeneity within the patient population. Therefore, there exists a great need for a more generalized approach to blocking the reactivation of MAPK signaling during treatment with KRAS^G12C^ inhibitors. Because many of the stress-adaptive mechanisms described above are activated through MAPK signaling, it is likely that they also play a role in resistance to KRAS^G12C^ inhibitors. Thus, investigating the role of stress-adaptive mechanisms in this process may provide insight into strategies to prevent and overcome emerging resistance to KRAS^G12C^ inhibitors. Overall, as new therapies arise that aim to target oncogenic RAS, the integrated stress response of the cell should be considered in terms of investigating resistance mechanisms, combining therapies and identifying biomarkers, in order to block resistance and enhance patient outcomes.

## Conclusions

The findings we discuss here indicate that although tumor resistance is multifactorial, stress-adaptive mechanisms might provide key targetable vulnerabilities in RAS-driven tumors. From a therapeutic perspective, the combinatorial inhibition of RAS, its downstream signaling pathways, multiple stress-response pathways and/or adaptive mechanisms to pan-stress stimuli, provide a promising approach to the treatment of these tumors. Perhaps, these combinations could be stratified based on which stress-response pathways are known to be activated among different RAS-driven cancers or as a result of RAS-targeted therapies. The upregulation of stress-response pathways might also be used as biomarkers of resistance, as well as of responses to specific therapies. Therapies that have previously failed in the clinic might also regain clinical traction, particularly once the stress-adaptive pathways or proteins that aid in a specific resistance mechanisms to a therapy are identified. It is exciting to consider the possibility that RAS-driven stress-adaptive mechanisms could provide a promising new avenue of investigation for therapeutics that alone or in combinations could successfully treat RAS-driven cancers.

This article is part of a collection ‘The RAS Pathway: Diseases, Therapeutics and Beyond’, which was launched in a dedicated Special Issue guest edited by Donita Brady and Arvin Dar. See related articles in this collection at https://journals.biologists.com/dmm/collection/5089/The-RAS-Pathway.
